# Distribution of Uropathogens and Molecular Characterisation of Virulence Genes of Uropathogenic *Escherichia coli* (UPEC) in Catheter-Associated Urinary Tract Infections in Limpopo Province, South Africa

**DOI:** 10.3390/pathogens15080858

**Published:** 2026-08-18

**Authors:** Joy Maunye, Ivy Rukasha, Makwese Maepa, Onele Gcilitshana

**Affiliations:** 1 Department of Pathology and Medical Science, University of Limpopo, Turfloop Campus, Polokwane 0699, South Africa; ivy.rukasha@ul.ac.za (I.R.); makwese.maepa@ul.ac.za (M.M.); onele.gcilitshana@ul.ac.za (O.G.); 2 Department of Medical Microbiology, National Health Laboratory Services (NHLS), Polokwane 0699, South Africa

**Keywords:** *Escherichia coli*, catheter-associated urinary tract infection, virulence genes, Limpopo province

## Abstract

Catheter-associated urinary tract infections (CAUTIs) are among the most common nosocomial infections globally and represent a significant cause of morbidity and prolonged hospital stays. There is a scarcity of molecular data regarding pathogens and virulence factors linked to CAUTIs, particularly in South Africa. This study aimed to determine the distribution and frequency of uropathogens, including polymicrobial infection patterns, isolated from catheterised patients at the Polokwane National Health Laboratory Services (NHLS), Limpopo Province, from March 2024 to March 2025, and to characterise the prevalence and co-occurrence of six virulence genes (*fimH*, *papC*, *sfa*, *hly*, *cnf*, and *aer*) among *Escherichia coli* isolates, the predominant uropathogen identified. Specimens from catheterised patients diagnosed with urinary tract infections at the National Health Laboratory Services in Polokwane were used. Uropathogens were isolated on agar and identified using the VITEK^®^ system. Polymerase chain reaction (PCR) was used for amplification of some virulence genes (*fimH*, *papC*, *sfa*, *hly*, *cnf*, and *aer*) associated with uropathogens. Confirmation of urinary tract infections revealed that *Escherichia coli* was the predominant uropathogen (32.8%), followed by *Klebsiella pneumoniae* (17.8%), *Enterococcus faecalis*, *Enterococcus faecium* (14%), and others. Among *E. coli* isolates, virulence gene analysis revealed *fimH* (79.8%) as the most prevalent, followed by *aer* (77.4%), *sfa* (41.7%), *hly* (27.4%), *papC* (26.19%), and lastly *cnf* (9.5%). This study demonstrates that *E. coli* is the leading cause of CAUTIs in our study population, exhibiting diverse virulence gene combinations that enhance adhesion and overall pathogenicity. The findings emphasise the need for routine molecular surveillance of CAUTI pathogens, reinforcement of catheter-care protocols, and targeted infection prevention strategies.

## 1. Introduction

Urinary tract infections (UTIs) represent a significant proportion of infections encountered in both healthcare institutions and community settings, making them one of the most prevalent infectious disease entities [1]. To date, urinary tract infections (UTIs) affect more than 400 million people globally each year [2]. The prevalence of UTIs in Sub-Saharan Africa has progressed from 32.12% [3] to a prevalence as high as about 67% [4]. Studies show that approximately 50% of adult women may experience a urinary tract infection (UTI) more than once throughout their lifetime. This high recurrence rate highlights the importance of understanding the risk factors and preventive measures associated with UTIs, especially in this demographic [5,6].

Catheterization is a major contributor to the onset of complex urinary tract infections (UTIs), particularly giving rise to CAUTIs. Catheterization remains one of the most common causes of nosocomial infections, highlighting their prevalence and clinical relevance in patient care settings [7]. As a result, about 75% of UTIs developed in hospitals are associated with a urinary catheter [8,9]. These infections occur after catheter insertion in a patient for more than 48 h, with symptoms like a rise in body temperature, suprapubic tenderness, or costovertebral angle tenderness [10].

A wide range of microorganisms have been implicated in CAUTIs, but *Escherichia coli* remains the predominant etiological agent worldwide, responsible for approximately 30–50% of cases [11]. Other significant pathogens include *Enterococcus faecalis*, *Klebsiella pneumoniae*, *Proteus mirabilis*, *Pseudomonas aeruginosa*, and *Enterobacter* species. These organisms are often linked with biofilm formation on catheter surfaces, enabling persistence within the urinary tract and resistance to host immune responses and antimicrobial therapy [12,13,14,15,16].

One of the crucial techniques that contributes to a better understanding of causative agents of CAUTIs at the genomic level is molecular characterisation [17]. Multiplex Polymerase Chain Reaction (mPCR) is a highly valuable technique in the field of molecular characterisation [18]. Most uropathogens possess a wide range of virulence genes encoding virulence factors that enhance their ability to defeat different defence mechanisms to cause an infection [19].

Expression of some of these virulence genes encoding virulence factors contributes to disease severity by enhancing pathogenesis and antimicrobial drug resistance. This leads to substantial medical costs and increased mortality [20]. Thus, determining the virulence factors of common uropathogens is key to making the most accurate diagnosis, tracing the source of infection, choosing appropriate treatment, and overall understanding of their epidemiology and pathogenesis. There are limited reports on molecular characterisation of UTIs, especially CAUTIs in South Africa. This study therefore aimed to: (1) determine the distribution and frequency of uropathogens, including polymicrobial infection patterns, isolated from catheterised patients attending healthcare facilities across Limpopo Province whose specimens were processed at the Polokwane NHLS Medical Microbiology Laboratory, over a one-year period; and (2) characterise the prevalence and co-occurrence patterns of six virulence genes (*fimH*, *papC*, *sfa*, *hly*, *cnf*, and *aer*) in *Escherichia coli* isolates, as the predominant uropathogen, using polymerase chain reaction (PCR).

## 2. Materials and Methods

### 2.1. Study Design

This study employed an observational, descriptive, cross-sectional design. Secondary data were obtained from the NHLS Medical Microbiology Laboratory over a one-year period. The study did not involve direct contact with participants; specimens were residual catheter urine samples submitted by healthcare providers across Limpopo Province. Ethical clearance was obtained from the Turfloop Research Ethics Committee (TREC/1583/2024: PG), and patient informed consent was waived given the non-direct contact with patients. For the purposes of this study, a CAUTI was operationally defined in accordance with CDC/NHSN criteria as the presence of at least one sign or symptom (fever > 38 °C, suprapubic tenderness, costovertebral angle pain or tenderness, dysuria, or urinary urgency/frequency) with a positive urine culture yielding ≥10^3^ CFU/mL of no more than two uropathogen species, in a patient with an indwelling urinary catheter in situ for ≥48 h at the time of specimen collection [21]. The study did not have permission to access the patient’s file; clinical data (patient history, diagnoses, catheter duration, treatments, etc.) could not be obtained.

### 2.2. Study Population

The study focused on samples from previously catheterized patients presumed to have urinary tract infections based on their symptoms. The study featured all patient samples from any department/area (medical, surgical, gynaecological, etc.) at over 250 hospitals, clinics, and other healthcare services across Limpopo Province sent to the National Health Laboratory Services (NHLS). Polokwane NHLS (Medical Microbiology Laboratory) serves the entire province with culturing and interpretation of UTI samples from public healthcare providers.

### 2.3. Data Collection

A total of 215 samples were obtained from the Polokwane NHLS over a one-year period, from March 2024 to March 2025. Demographic data were collected from the NHLS request form using a data collection tool.

### 2.4. Inclusion and Exclusion Criteria

#### 2.4.1. Inclusion

All urine specimens submitted to the NHLS Polokwane from catheterised patients deemed probable CAUTIs by the doctor/clinician/healthcare provider from any public healthcare facility in Limpopo Province during the study period were eligible. Specimens were included if they yielded significant bacteriuria, defined as growth of ≥10^3^ colony-forming units (CFU)/mL of a recognised uropathogen on chromogenic agar. Samples from all departments (medical, surgical, paediatric, gynaecological, neonatal ICU, etc.) and all patient age groups were included.

#### 2.4.2. Exclusion

Specimens were excluded if they showed no growth or growth below the diagnostic threshold (<10^3^ CFU/mL), if they had insufficient volume for a culture or if the accompanying request form lacked sufficient demographic data (age, gender, or facility of origin) for analysis.

### 2.5. Disposal Criteria for Isolates

Discarded isolates were placed in designated, puncture-proof, leak-proof, tightly sealed, red biohazard containers and collected by a licenced biohazardous waste collector for further decontamination and disposal of the waste.

### 2.6. Laboratory Tests and Procedures for Data Collection

#### 2.6.1. Isolation of the Most Common Uropathogen

A loopful of urine was delivered onto a chromogenic agar plate by making a straight line down the plate with the same loop. The agar plate was streaked out from the primary inoculation pool from side to side in a downward movement. The plates were incubated for 18–24 h in the walk-in aerobic incubator. The colonies on the chromogenic media were interpreted according to the manufacturer’s instructions based on colour and morphology.

#### 2.6.2. Identification and Confirmation of Isolates

Isolates were identified by the Vitek MS Prime Mass Spectrometry Microbial Identification System (bioMérieux, Marcy-l’Étoile, France), which uses MALDI-TOF technology for rapid identification. It can be divided into four steps/parts, as follows: Sample preparation, where bacterial or yeast cells from a culture are deposited onto a disposable, barcoded target slide using the VITEK MS Prep Station. This is followed by matrix addition, where a ready-to-use matrix solution is added to the sample spots. Next is the analysis, wherein the target slide is loaded into the VITEK MS acquisition station, and a laser generates protein mass spectra, which are analysed by the system’s Advanced Spectra Classifier algorithm. Lastly, result generation, wherein the system compares the generated spectrum against a comprehensive reference database to provide an organism identification with a confidence value.

#### 2.6.3. Detection of Virulence Genes of UPEC

##### Bacterial DNA Extraction

Bacterial DNA was extracted using the KingFisher™ Flex Purification System (Thermo Fisher Scientific, Waltham, MA, USA), with a 96 Deep-well Head. MVP_2wash_200 flex protocol and MagMAX™ Viral/Pathogen Nucleic Acid Isolation Kit (Thermo Fisher Scientific, Waltham, MA, USA) were used on the system. The BindIt software 4.1 (version 4.1.0.2) was used to carry out extraction on the KingFisher™ Flex Purification System.The extraction was divided into four steps. First step: setup of the KingFisher instrument. The instrument was set up with a proper magnetic head (96 deep-well magnetic head) and the proper heat block (96 deep-well heat block), and the MVP_2wash_200 flex protocol was downloaded and loaded onto the instrument. Second step: setup of the processing plates. The Wash, Elution, and Tip comb plates were set up outside of the instrument. Third step: preparation of a binding mixture; 265 µL of binding solution was mixed with 10 µL of total nucleic acid magnetic beads and mixed well by inversion. Fourth step: digestion with proteinase K and elution of nucleic acid. Then, 10 µL of proteinase K was added to each well of a 96 deep-well sample plate. Next, 200 µL of 0.5 McFarland (sample) from the culture plate colonies was added to wells with proteinase K in the sample plate. Then, 550 µL of the Binding bead mix was then added to each sample in the sample plate. The MVP_2wash_200 flex protocol was selected on the instrument, and the run was started. The prepared plates were loaded into position when prompted by the instrument. After the protocol was completed, the Elution plate was immediately removed from the instrument and covered and stored at −20 °C, and was ready to use for PCR. For extraction control(s), a no-template extraction blank (nuclease-free water substituted for sample) was included in every extraction batch to detect reagent or environmental contamination of the extraction process.

##### Primer Sequences

The virulence genes examined in this study include those responsible for type 1 fimbriae (*fimH*), which enable adhesion, biofilm formation, and persistence on catheter surfaces [22], pyelonephritis-associated pili (*papC*) that help in bacterial adhesion to glycolipid receptors on kidney epithelial cells, promoting ascending infections like pyelonephritis [23,24], S and F1C fimbriae (*sfa* and *foc*), which are surface structures that enable bacterial adhesion to host cells [25], hemolysin (*hly*), which is a key virulence determinant, as a pore-forming toxin that damages host cells [25,26], cytotoxic necrotizing factor (*cnf*), which aids in bacterial persistence and immune evasion [25], and aerobactin (*aer*), which enables iron acquisition in iron-limited environments like the urinary tract [27]. The selection of these virulence genes is grounded in the existing literature, as they grant UPEC fitness to cause UTIs and are among the most commonly investigated, with various studies exploring their roles [19,28,29,30,31,32,33,34,35,36,37,38,39].

Comprehensive details regarding the specific primer sequences and the expected sizes of the amplified products are provided in Table 1. Detection of *fimH*, *papC*, and *sfa* sequences was done by multiplex PCR, as in Dadi et al. (2020) [19] and Tarchouna et al. (2013) [29], while *hly*, *cnf*, and *aer* were detected by single-plex PCR.

##### Amplification

Amplification of the bacterial DNA was done in a total volume of 46 µL containing 20 µL of nuclease-free water, 20 µL of 2XPhusion™ Plus Green PCR Master Mix (the mixture contains DNA polymerase, salts, magnesium, and dNTPs for efficient PCR amplification), 2 µL of DNA template, and 2 µL (15 µM) of each of the primers, giving a final primer concentration of 0.652 µM per primer. The cycling conditions for each gene are shown in Table 2. The amplification was carried out in a SimpliAmp™ Thermal Cycler (Thermo Fisher Scientific) with a maximum ramp rate of 4 °C/s. (block), 3 °C/s. (sample), thermal range of 0 °C to 100 °C, and thermal uniformity of <0.5 °C (20 s. after reaching 95 °C). All PCRs were performed for 35 cycles following the initial denaturation step (Table 2). Positive and negative controls: For *fimH*, *papC*, *sfa*, and *aer*, ATCC 35218 was used as a positive control. For *sfa*, *papC*, and *hly*, ATCC 25922 was used as an additional positive control. For *cnf*, one positive sample isolate was selected during trial/optimisation runs and was used as a positive control for the *cnf* runs, as the ATCC strains used as controls in this study did not carry the *cnf* gene. For negative control, nuclease-free water was included in every run as a no-template PCR negative control. To validate controls, any PCR run in which the positive control failed to amplify or the negative control showed amplification was considered invalid, and the entire run was repeated before reporting the results.

##### Visualisation

To observe the amplification of DNA, a 10 µL aliquot of the PCR product was loaded on 2% agarose gel for electrophoresis, stained with 30 µL of SYBR™ Safe DNA Gel Stain or 15 µL of Ethidium bromide. Electrophoresis was carried out for 80 min at 200 volts on the 1XTBE/TAE buffer system, and the gel was visualised under the iBright™ CL750 Imaging System (iBright™ CL750 Imaging System). Amplified DNA fragments of specific sizes were detected, and the sizes of the amplicons were estimated by comparing them with the 100 or 50 bp plus DNA ladder (Invitrogen™, Thermo Fisher Scientific, Waltham, MA, USA) included on the same gel.

### 2.7. Statistical Analysis

All data were recorded on Microsoft Excel and analysed using SPSS version 28. Descriptive statistics were used to summarise demographic variables. Associations between demographic variables (age, gender, race) and uropathogen isolation rates and co-infection patterns were assessed using the Pearson chi-square test where all expected cell frequencies were ≥5, and Fisher’s exact test where any expected cell frequency was <5. Odds ratios (ORs) with 95% confidence intervals (CIs) were calculated for all associations.

## 3. Results

Unless otherwise specified, pathogen distribution frequencies are reported as proportions of total isolates (*n* = 255); demographic proportions are based on total patients (*n* = 215); and *E. coli*-specific analyses, including co-infection patterns and virulence gene prevalence, are expressed as proportions of *E. coli* isolates (*n* = 84).

### 3.1. Demographic Status of Patient Samples

**Figure 1:**
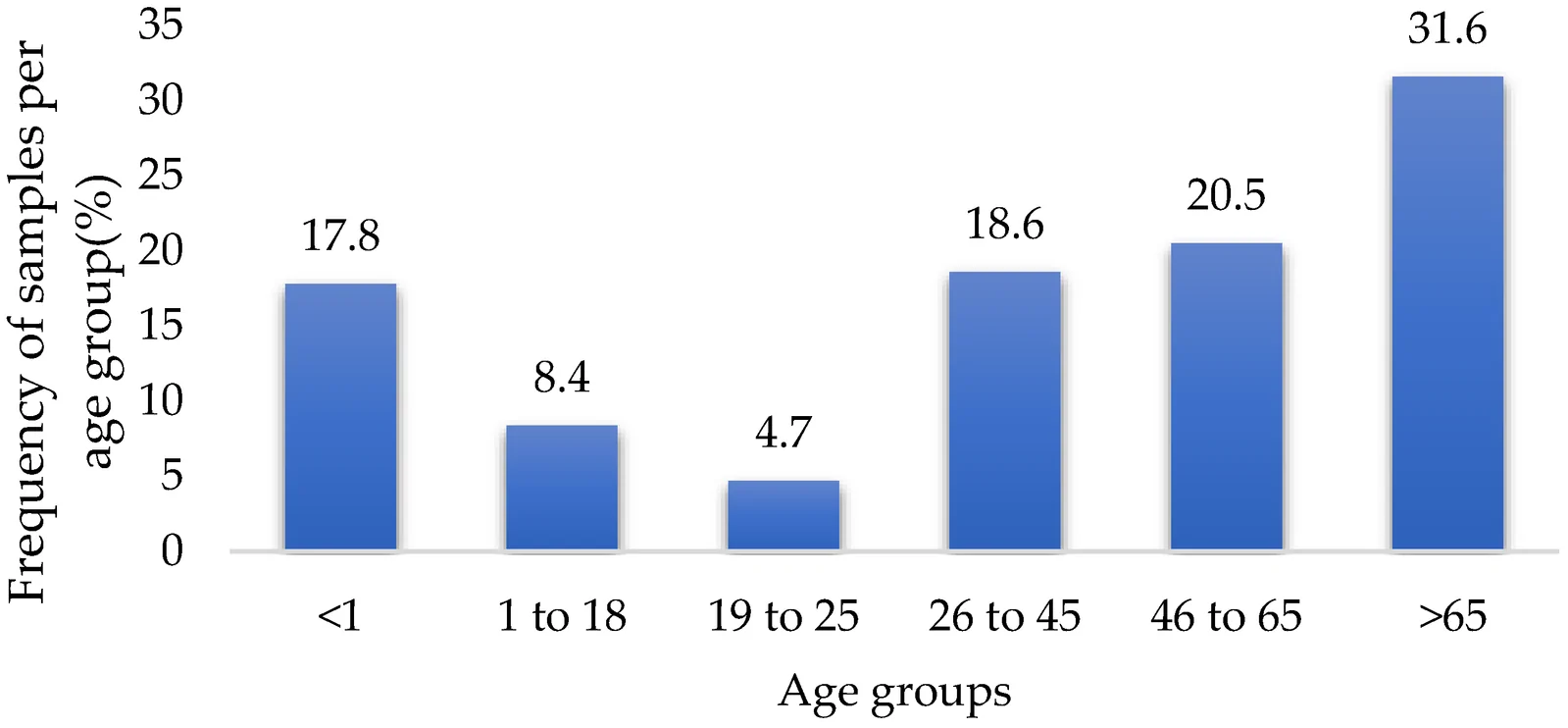
Distribution of study participants with CAUTI samples from March 2024 to March 2025 by age group.

**Figure 2:**
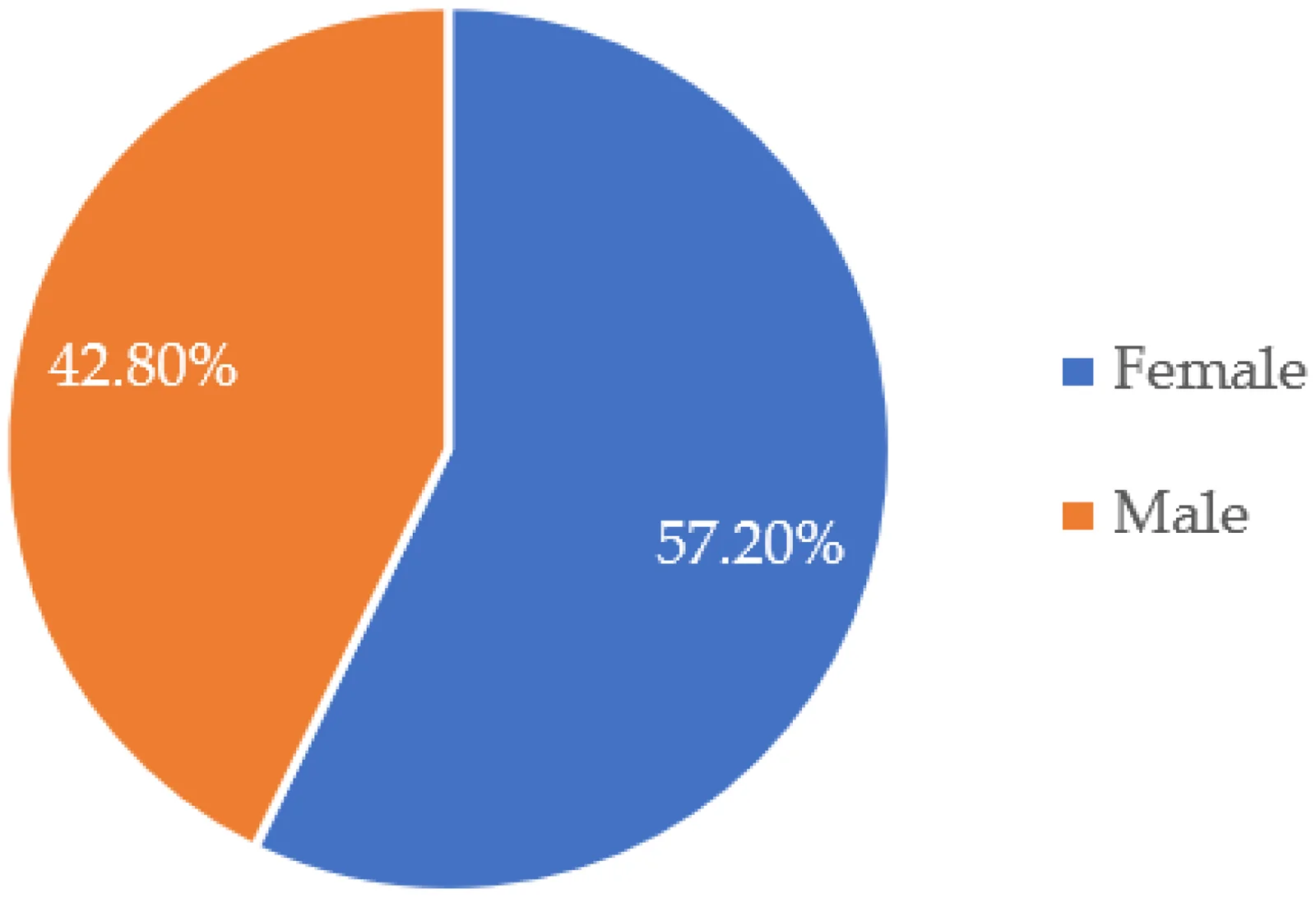
Distribution of study participants with CAUTI samples from March 2024 to March 2025 by gender.

**Figure 3:**
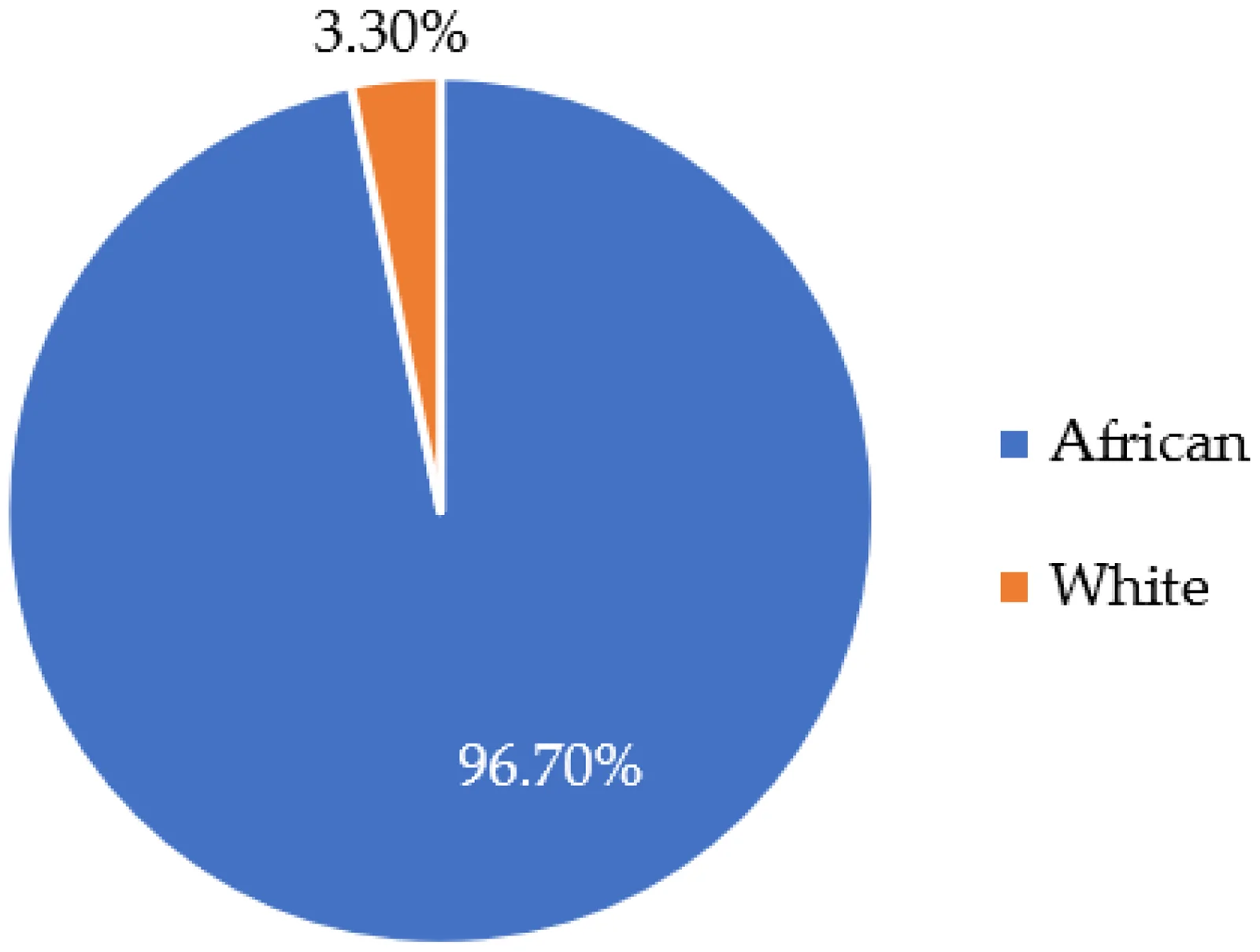
Distribution of study participants with CAUTI samples from March 2024 to March 2025 by race.

A total of 215 patient samples from CAUTI patients who were catheterized from different public healthcare facilities across the entire Province were investigated in this study. Samples consisted of individuals from all age groups, wherein most were from participants aged >65 years (*n* = 68), followed by those aged 46–65 years (*n* = 44), then the 26–45 year (*n* = 40), <1 year (*n* = 38), 1–18 years (*n* = 14), and 19–25 years (*n* = 10) age groups, respectively. The mean age of the study participants was found to be 44.54 years, with a range of 0 to 98 years. We also noted that of all these samples collected, *n* = 123 were females and *n* = 92 were males. In terms of race, it was found that *n* = 208 of the participants were African and *n* = 7 were white. These demographics are shown in Figure 1, Figure 2 and Figure 3 below.

### 3.2. Distribution of Uropathogens Isolated from the 215 CAUTI Samples over a One-Year Period

**Figure 4:**
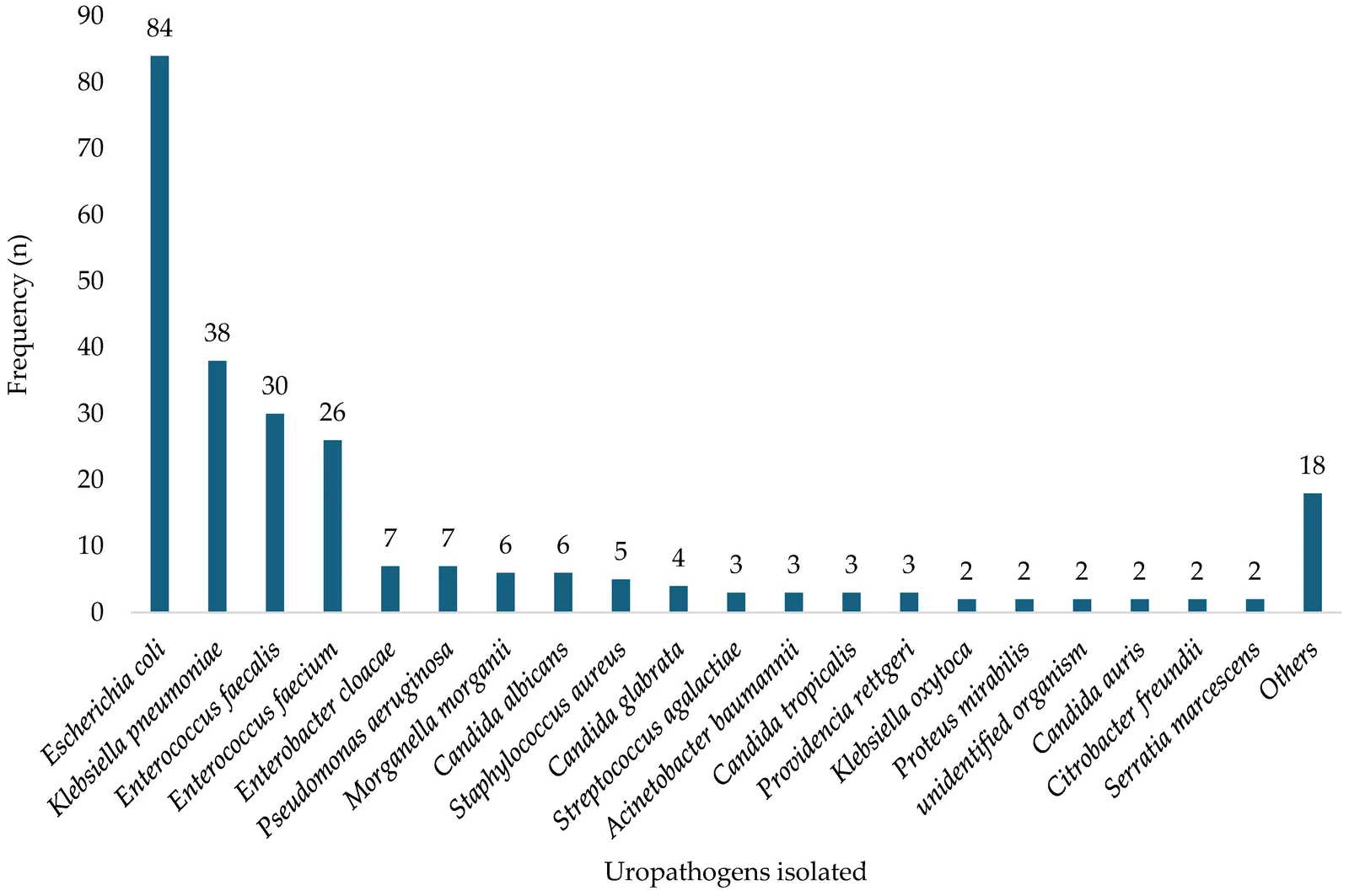
Distribution of uropathogens isolated from the 215 CAUTI samples over a one-year period.

A total of 255 microorganisms were isolated. The most prevalent microorganism was *E. coli*, with 84 occurrences, followed by *K pneumoniae* (*n* = 38), *Enterococcus faecalis* (*n* = 30), and *Enterococcus faecium* (*n* = 26). Some pathogenic yeasts (*Candida* spp.) were also identified, though in a smaller fraction; these include *Candida* albicans (*n* = 6), *Candida* auris (*n* = 5), *Candida glabrata* (*n* = 4), and *Candida tropicalis* (*n* = 3); see Figure 4. Among the 84 *E. coli* isolates, *n* = 60 (71.4%) were single *E. coli* infections, while n = 24 (28.6%) were part of *E. coli* co-infection/polymicrobial infections. The 24 polymicrobial infections included *n* = 12 (14%) co-infections of *E. coli* + *E. faecalis*, *n* = 6 (7%) of *E. coli* + *K. pneumoniae*, and *n* = 6 (7%) with other uropathogens.

#### Association of *E. coli* Infection Rate and Co-Infection with Demographic Variables of CAUTI Patients from Limpopo Province

The chi-square statistical test revealed there was a significant association between *E. coli* rate and gender among the 215 samples, with female participants having a higher *E. coli* isolation rate than male participants, and it was also found that *E. coli* isolation rate was statistically associated with age, with the elderly age group showing a higher rate of *E. coli* isolation relative to other age groups. Fisher’s exact test revealed that there was no significant association between race and *E. coli* rate, as shown in Table 3 below.

The chi-square statistical test revealed there was no significant association between *E. coli* co-infection rate and gender, age among the 84 isolated *E. coli*. Fisher’s exact test revealed a non-significant association between age and co-infection rate. Refer to Table 4 below.

### 3.3. Detection of UPEC Virulence Genes

**Figure 5:**
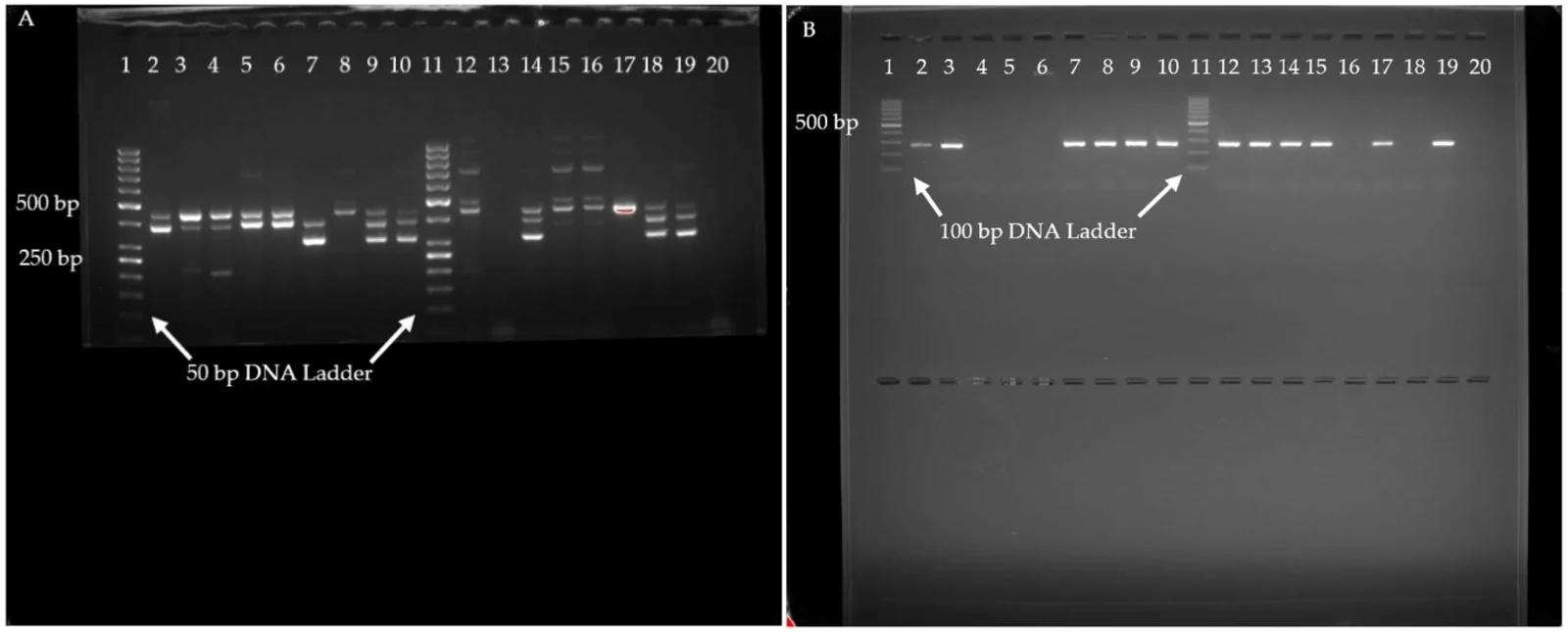
Representative 2% agarose gel electrophoresis of uropathogenic *E. coli* virulence genes isolated from catheter-associated urinary tract infection patients. (**A**) Lane 1 and lane 11 (L1 and L11) represent a 50 bp DNA ladder; lanes L2–L8, and L12–L17 represent amplified *E. coli* PCR products (samples 1–13) for the following virulence genes: *papC* (328 bp), *sfa* (410 bp), and *fimH* (456 bp); lane 9 (L9) represent positive control 1 (ATCC 35218); lane 10 (L10) represent another positive control (ATCC 25922); and lane 20 (L20) represents the negative control (nuclease-free water). (**B**) Lanes 1 and 11 (L1 and L11) represent a 100 bp DNA ladder; lanes L2–L8 and L12–L19 represent amplified *E. coli* PCR products (samples 1–16), detecting the *aer* (269 bp) gene. Lane L10 represents the positive control (ATCC 35218); lane L20 represents the negative control (nuclease-free water).

The virulence genes *fimH* (456 bp), *papC* (328 bp), *sfa* (410 bp), *aer* (269 bp), *cnf* (693 bp), and *hly* (556 bp) were successfully amplified. Figure 5 below shows gel pictures representing the amplification of some genes.

**Figure 6:**
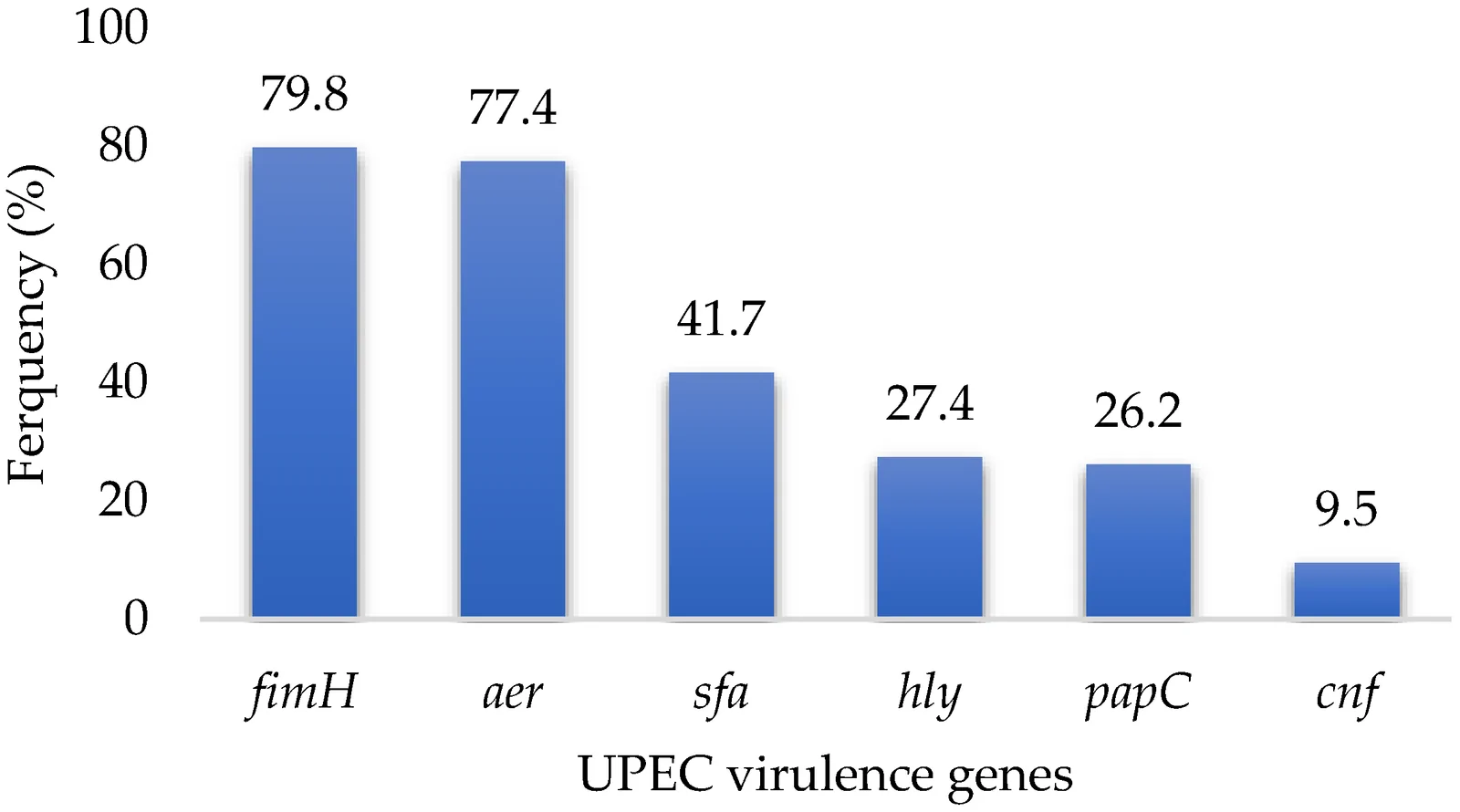
Distribution of virulence genes.

Six virulence genes that are among the most common UPEC virulence genes were tested. The study found that *fimH* was the most frequent, with a prevalence of *n* = 67 positive isolates out of a total of 84 *E. coli* isolates, followed by *aer* *n* = 65, *sfa* *n* = 35, *hly* *n* = 23, and *papC* *n* = 22, and the least frequent was *cnf* *n* = 8. Refer to Figure 6 below.

The most common gene co-occurrences (combinations) observed from the 84 isolated UPEC infections were 51 *fimH* + *aer*, 30 *fimH* + *sfa*, and 28 *aer* + *sfa* (Table 5). Only one isolate was negative for all the genes, and one isolate was positive for all six genes.

## 4. Discussion

### 4.1. Demographic Status of Participants

This study found a high incidence of CAUTIs in women, who accounted for 57.2% of the participants, and men 42.8%. Many researchers have reported similar findings to ours [40,41,42,43,44,45,46,47]. These findings may be attributed to the female sex being a risk factor for multiple reasons. Among these reasons is the anatomical difference between both genders, wherein the female urethra is located closer to the anal opening, making it easier for bacteria, primarily UPEC and other enteric bacteria from the gastrointestinal tract, to colonise the periurethral area and enter the urethra [5,48,49,50]. Another popular reason may be that women have a significantly shorter urethra than men, which leads to the bacteria travelling a shorter distance to reach the bladder [19,42,43].

This study found that the age group of patients >65 years had the most CAUTI incidences. Numerous studies are consistent with our findings [43,51,52]. This may be due to multiple reasons, such as prolonged catheterization (older adults, especially those with chronic or complex conditions, often require indwelling catheters for longer periods, which is the most significant risk factor for CAUTIs and reduced immunity. As people age, their immune system becomes less effective, making it harder to fight off infections. These and other individual factors make them more susceptible to the bacteria that can cause a CAUTI, and a higher prevalence of chronic comorbidities, which are more common in older age, such as diabetes, immobility, and dementia, thus increases the risk of CAUTIs [37,46].

Another interesting observation from the age groups was the high number of babies who were less than one year old, accounting for 17.4% of CAUTI incidences. There are various studies with similar findings [47,48,49,50,51]. This may be due to neonates and young infants often requiring indwelling urinary catheters for extended periods due to prematurity, congenital anomalies, surgical interventions, or monitoring in Neonatal ICUs (NICUs) [52,53]. Infants have weak/underdeveloped immune responses, making it harder to clear pathogens efficiently. Additionally, their shorter urethra (especially in females) facilitates easier bacterial entry from the perineal flora [10,54]. Additionally, diapered infants are prone to faecal–perineal contamination, which can introduce uropathogens directly to the catheter site. Studies link prolonged diaper exposure to higher UTI risk, as moisture and bacteria from stool amplify colonisation, particularly in resource-limited or high-volume care settings [55].

African people made up 96.7% of the study participants, while the remaining 3.3% were white. This disparity in race makes sense because Limpopo is a rural province predominantly with black people with low economic means who rely on public healthcare services; on the other hand, the minority white population mostly have the economic capacity to afford private healthcare [56,57,58].

### 4.2. Distribution of Isolated Microorganisms/Isolation and Identification of Uropathogens

In the literature, the most common causative agent is *E. coli*, followed by other members of the Enterobacteriaceae group [5,14,23,45]. The findings of this study corroborate those in the literature, as the most prevalent pathogen was *E. coli*, followed by other Gram-negative bacteria and fewer occurrences of some yeasts, specifically *Candida* species of known nosocomial occurrence. From the 215 positive samples, *E. coli* *n* = 84 (32.8%) was the most common isolated microorganism, which is consistent with occurrences in studies from across the globe; in Tanzania, for instance, prevalences of 30.7% and 33.1–43.7% have been reported [44,59], and in Pakistan, a prevalence of 37% [60] has been reported. However, studies with a lower prevalence relative to this study were also noted, such as studies in India, with a prevalence of 22% [61], Sierra Leone, with a prevalence of 23% [62], and Poland, with 25.81% [63]. Local studies with a higher prevalence relative to this present study were noted, with prevalences of 54.2%, 81.25%, and 57.6% [4,64,65]. Other studies with prevalence higher than the present study include studies from Lebanon (44.8%) [14], India (56.40%) [15], Iran (62%) [66], and Sweden [67].

This study found *Klebsiella pneumoniae* to be the second leading uropathogen, followed by *Enterococcus faecalis*, *Enterococcus faecium*, and *Pseudomonas aeruginosa*. This is in line with studies from South Africa [65], Uganda [68], Central Europe [69], Bulgaria [70], India [46], Iran [66], and Indonesia [40]. This pattern is consistent with reports indicating that *K. pneumoniae* is a major cause of hospital-acquired UTIs, particularly in catheterised patients, owing to its strong biofilm-forming ability and widespread antimicrobial resistance [11]. The detection of *Enterococcus faecalis* and *Enterococcus faecium* as subsequent leading pathogens is also consistent with the literature describing Enterococci as important nosocomial uropathogens capable of persisting in the hospital environment and colonising indwelling devices [71]. Although *Pseudomonas aeruginosa* occurred at a lower frequency, its presence is clinically relevant, as it is often associated with catheter use and increased risk of multidrug resistance in hospital settings. However, some studies report differing pathogen distributions, with *P. aeruginosa* or Enterococci ranking higher depending on catheter duration, ICU admission, antimicrobial pressure, or institutional microbiological trends, illustrating the context-specific nature of CAUTI epidemiology [72]. The pathogen profile observed in this study reflects typical CAUTI-associated microbiology while highlighting the need for continued surveillance across diverse clinical settings.

The high rate of isolation of *E. coli* may be due to various factors, such as patient demographics and population characteristics. Higher *E. coli* rates often occur in studies focused on specific high-risk groups where UPEC thrives due to host vulnerabilities, such as shorter urethras in females or impaired immunity in certain comorbidities [41]. For instance, outpatient or community-onset cohorts emphasise uncomplicated-to-complicated transitions, where *E. coli* dominates (up to 65% in complicated UTIs), unlike our samples, where patients have mostly been hospitalised, and participants include diverse inpatients (i.e., elderly, ICU, etc) prone to polymicrobial growth, diluting *E. coli* proportions [53]. Elderly populations skew rates of occurrences; studies show that *E. coli* prevalence drops 10–20% in elderly-heavy cohorts due to the presence of competing Gram-positives [73,74,75].

Most samples used in our study represent elderly and paediatric patients, who mostly require long-term catheterisation due to their age-related complications. Laboratory methods, definitions, and mixed infections can also be used to potentially explain the differences between our study and other studies. Differences in culture media, cutoffs for positivity, whether mixed infections were counted, and whether molecular methods were used can affect percentages [76,77]. In our study, we counted 24 mixed infections out of 84 *E. coli* infections, and other studies may not have considered mixed infections or used different culture interpretation rules/procedures from ours, which might have led to the difference in *E. coli* isolation rates.

A chi-square test revealed a significant association (0.025) between gender and the *E. coli* isolation rate, with the female gender proving to be a risk factor for *E. coli* isolation, ultimately leading to CAUTIs. This is in line with studies from China, *p* < 0.001 [78,79], Italy, *p* < 0.001 [80], North Macedonia, *p* = 0.000 [81], and the USA, *p* = 0.0093 [27]. This association further strengthens the notion that women are more prone to UTIs because of their anatomy, that is, their shorter urethra and the proximity of their anal region to the periurethral area.

A significant association was also observed between patient age and *E. coli* isolation rate (*p* < 0.001), with the highest prevalence noted in individuals aged above 65 years. This suggests that *E. coli* isolation may be dependent on age. This observation aligns with reports/studies from Italy *p* = 0.0005 and *p* < 0.05 [80,82], China, *p* < 0.001 [53], Saudi Arabia and Indonesia, where a high *E. coli* isolation rate was observed in the elderly [83,84]. This further supports the conclusion that advancing age may predispose individuals to *E. coli*-driven CAUTIs, most likely due to age-related factors such as declining immunity, comorbidities, and prolonged catheterisation [53,85]. Race was found to be statistically insignificant when associated with *E. coli* isolation rate. This may have been due to the large disparity in our racial distribution.

#### UPEC Co-Infection Rate

Our study found an *E. coli* isolation co-infection rate of 28%, which, when compared to other studies from Iraq, 16.16% [86], the United Kingdom (UK), 68% [87], and Germany, 48.4%, shows a notable discordance. Possible reasons for this discordance might include factors such as population selection bias; if the study population is highly specific, such as patients living with comorbidities, the incidence of polymicrobial infections would be inherently higher compared to studies on the general population or healthy outpatients.

Diagnostic/detection methods also play a role in polymicrobial infections. Studies using advanced molecular techniques like PCR or next-generation sequencing can detect co-infecting organisms more readily than traditional culture-based and biochemical tests, which leads to lower polymicrobial rates. However, these are less economical and may not be a standard practice for hospitals in low-income countries. Our study used a traditional culture-based method because it was financially limited and therefore unable to access techniques such as those above. Some studies argue that sample size is also a factor; studies with larger sample sizes will tend to have higher rates than studies with lower sample sizes [88,89,90,91].

The *E. coli* and *E. faecalis* pair was found to be dominant in the *E. coli* co-infection rate. This observation aligns with a growing body of evidence highlighting *E. faecalis* as one of the most frequent co-pathogens recovered alongside *E. coli* in urinary and catheter-associated infections. A study by Salm and colleagues reported *E. faecalis* in approximately 47% of polymicrobial urine samples from male outpatients in Germany [92], while Nye et al. (2024) demonstrated that *E. coli* and *Enterococcus* frequently co-occur on indwelling catheter surfaces, forming robust mixed-species biofilms [93]. Similar findings have been described in earlier studies and experimental CAUTI models, where *E. faecalis* was shown to promote *E. coli* persistence and immune evasion within catheter biofilms [94]. These reports collectively indicate that the co-isolation rate observed in our cohort reflects a clinically and biologically plausible infection pattern.

The interaction between *E. coli* and *E. faecalis* in polymicrobial CAUTIs is clinically significant. Evidence suggests that *E. faecalis* can enhance the survival of *E. coli* by dampening host immune responses, contributing to chronicity and recurrence of infection [95]. From a therapeutic perspective, the presence of *E. faecalis* in mixed infections poses additional treatment challenges. *Enterococci* have intrinsic resistance to many β-lactams and aminoglycosides, limiting the efficacy of empiric regimens typically directed at Gram-negative organisms such as *E. coli*.

A statistically significant association between *E. coli* polymicrobial rate and age was also observed in this study, where more polymicrobial *E. coli* infections were observed in elderly individuals (aged > 65 years). This is suggestive of old age as a predictor of *E. coli* co-infections in CAUTIs. Our observation aligns with multiple recent investigations showing frequent polymicrobial catheter colonisation in older and long-term-catheterised populations, where *E. coli* commonly co-occurs with other urinary microbes [93]. Several recent reviews and epidemiologic analyses also report higher polymicrobial detection rates in older adults, an effect that is amplified when molecular diagnostics (i.e., M-PCR/metagenomics) are used, because these methods detect low-abundance cohabitants that routine culture may miss [96]. However, some institution-level datasets and adjusted multivariable analyses have failed to find age as an independent predictor once factors such as catheter duration, prior antibiotics, comorbidity burden, and sampling/diagnostic methods are taken into account, implying that age may act largely through these mediating factors rather than as a direct causal driver [21]. Taken together, our results are consistent with a model in which advancing age and its correlates, such as long-term catheterisation, institutional residence, higher comorbidity and antibiotic exposure, increase the opportunity for polymicrobial colonisation, and more sensitive detection methods reveal this association more clearly [93]; we therefore recommend reporting catheter duration, prior antibiotic exposure, and the exact microbiologic identification method and discussing them as potential confounders.

### 4.3. Virulence Genes of UPEC

This section reports the observed frequency (prevalence) of virulence genes detected by PCR in the study cohort. It should be emphasised that the presence of a gene at the DNA level, as detected by PCR, does not equate to gene expression, protein production, or functional activity in vivo, nor does it establish the importance of that factor in the pathogenesis of infection in this population. Interpreting the role of these genes in adhesion, colonisation, or infection severity would require separate experimental studies, such as gene expression analysis, adhesion assays, and in vivo infection models, which were beyond the scope of this descriptive study. The contextual discussion of each gene’s known function is drawn from the peer-reviewed literature and is provided to frame the epidemiological significance of the observed prevalence, not to draw mechanistic conclusions from the present data.

This study observed the *fimH* gene as the most commonly present virulent gene, at 79.8%. This gene plays a significant role as a colonisation factor by binding to the urothelium during the manifestation of UTIs [97]. This is consistent with studies conducted in Iran, where they reported occurrences of 86.17% and 92.8% [28,31], Ethiopia, with an occurrence of 82% [19], India, with 87.5% [98], Mongolia, with 89.9% [20], and China, with 87.4% [99]. Our findings were noticeably different from recent UTI studies conducted in Sudan, which reported a prevalence of 63.6% [36], Tunisia, with 68% [29], and Iran, with 62.3% [33]. The discrepancy between our findings and those of other studies may potentially be due to different reasons, such as the populations sampled [36]. This study used convenience sampling, where all groups of patients were included, while other studies may have been more specific in terms of their study population, such as focusing on paediatrics, ICU patients, the elderly, etc. Sampling and selection bias might also be a factor. Studies that focus on MDR isolates, environmental isolates, or outbreak strains may under-represent/over-represent *fimH*-positive lineages [100]. Sample size and chance variation might also address the discordance in our findings with other studies; small studies tend to show greater random fluctuation in gene frequency than large surveillance cohorts [101].

In this study, *fimH* emerged as the most frequently co-occurring virulence gene, appearing in nearly all of the dominant gene combinations identified. The high prevalence of *fimH* reflects its fundamental role in UPEC pathogenicity, as type 1 fimbriae are essential for mediating adhesion to uroepithelial cells and indwelling catheter surfaces [11,31]. Its consistent co-occurrence with other virulence genes, particularly *aer*, *sfa*, and *papC*, might suggest that *fimH* forms a core adhesive backbone upon which additional pathogenic traits are built, but of course, this is outside of the study scope; appropriate tests should be done to further validate this. This pattern aligns with global findings showing that *fimH* is one of the most conserved and ubiquitous ExPEC virulence genes, providing a fitness advantage during urinary tract colonisation and persistence [102,103,104]. The dominance of *fimH*-containing combinations in this study therefore reinforces the central importance of adhesion as a primary strategy used by CAUTI-associated *E. coli* to initiate and maintain infection in catheterised patients.

S and FIC (*sfa* genes) were found in 35 (41.7%) of the UPEC isolates. These findings are in line with those of studies from Tunisia (34%) [29], India (49% and 39.1%) [105,106], and Iran (48.46%) [107]. However, studies from Ethiopia (25%) [19], Iran (79.4%, 13.6%, and 81%) [30,32,34], Egypt (29.3%) [35], and Taiwan (29.8%) [38] are discordant with our study. Studies report that S fimbriae have abundant receptors in the bladder and the epithelial surface of the kidneys. This contributes to the easy ascension of UPEC to the kidneys, causing pyelonephritis, which forms one of the serious, complicated UTIs [108,109,110]. This study observed 41.67% of *sfa* genes from the isolated UPEC infections; this means about 41% of patients who were positive for UPEC were potentially at risk for ascending UTIs, leading to nephritis. The *sfa* genes have previously been detected in neonatal meningitis and septicaemia [37,111]. This also suggests that neonates who were positive for UPEC had a potential risk of meningitis and septicaemia. There was no significant association between *sfa* genes and gender, age, and race.

The pyelonephritis-associated pili (*papC*) gene was found in 22 (26.2%) UPEC isolates, which can be compared to studies from Iran, (16.7%) [112], Mongolia (20.3%) [20], Ethiopia (29.5%) [19], and Iran (27.1%) and (32%) [113,114]. However, it is discordant with studies from Tunisia, (41%) [29], Iran (61.3%) [115], Egypt (52%) [35], Indonesia (65.85%) [116], and Iran (91%) [117]. The discordance between our study and other studies may be due to multiple explainable causes. Studies enriched for upper-tract infections or biofilm/catheter isolates often show a much higher prevalence of *papC*. Conversely, studies of mixed outpatient cystitis or commensal collections report lower *papC* [110]. Therefore, population and study setting may have played a role in this case. Some studies report that the prevalence of *papC* genes tends to be higher in strains isolated from severely ill patients and those with pyelonephritis, and *E. coli* harbouring *papC* genes are more frequent, particularly in inpatient settings and severe infections, compared to less severe outpatient cases [5,118,119]. Additionally, the *papC* gene is enriched in certain phylogroups, such as in many B2 strains, and may be absent or disrupted in other successful lineages such as some ST131 clones; this lowers the overall *papC* frequency in collections dominated by those lineages [120,121].

As already indicated in the present study, the *papC* gene was detected in 26.2% of the *UPEC* isolates. This prevalence indicates that a substantial minority of isolates carry P fimbriae, which are well-documented virulence determinants associated with upper urinary tract infections and pyelonephritis [110]. P fimbriae mediate adhesion to the renal tubular epithelium and contribute to bacterial persistence and host tissue damage [122,123]. Their detection in approximately one-quarter of isolates suggests that while some strains possess strong kidney-tropic potential, many potentially rely on other adhesins such as *fimH* and *sfa* to establish infection, reflecting the heterogeneity of uropathogenic *E. coli* (UPEC) circulating in the study area.

From a clinical perspective, the moderate *papC* prevalence observed here implies that a subset of infections in this cohort could possess enhanced potential for ascending or recurrent disease, but most CAUTI cases are likely caused by isolates expressing other adhesins. These findings underscore the importance of evaluating multiple virulence factors rather than relying on a single genetic marker to characterise pathogenic potential in UPEC populations. There was no statistical significance between the *papC* gene and the demographic variables.

In this study, 23 (27.4%) UPEC isolates had the hemolysin (*hly*) gene, which is comparable to studies from Poland (22%) [124], Iran (25%) and (31%) [114,125], Iraq (21.9%) [126], Nepal (32.3%) [127], and South Korea (20.3%) [128]. Studies with discordant results were noted in China (8.33%) [129], Syria (41.6%) [130], Ethiopia (51.5%) [19], and Mexico (15.4%) [131]. The discordance of our results with some of the published articles may be attributed to different plausible factors, such as local strain population (Phylogroup or Sequence Type (ST) and laboratory methods for the detection of the *hly* genes (different PCR targets/primers, different PCR protocols) [129]. Because *hlyA* is frequently associated with certain UPEC lineages, notably phlyogroup B2, the observed prevalence likely reflects the mix of sequence types in our catheter-associated collection and the healthcare setting. We therefore recommend that readers interpret the 27.4% figure in the context of these factors (geography, catheter-associated vs. community isolates, and detection method).

The 27.4% prevalence of the *hly* gene in this study is within some published ranges and implies that a substantial minority of isolates may carry a potent cytolytic virulence factor (hemolysin), which is a pore-forming toxin associated with epithelial damage, immune evasion, and more severe disease phenotypes in experimental and clinical contexts. Strains that carry it are more likely to cause tissue injury or persistent infection [110]. That increases the clinical relevance of the isolates that carry *hly* in our study.

Long-term-catheterised and older/institutionalised patients commonly harbour polymicrobial biofilms and diverse UPEC lineages; prolonged catheter presence and healthcare exposures increase opportunities for colonisation by virulent lineages and for mixed infections to establish. This may justify the appearance of *hly* at this frequency in this CAUTI study [93].

This study observed a *cnf* prevalence of 9.5%, which is concordant with studies from Uganda (5.5%) [132], Iran (12.3%) [33], Mexico (14.02%) [133], and Tunisia (3%) [29], but studies with discordant results have been noted in Iran (50.4%) [134], Egypt (42%) and (38.09%) [135,136], and Iraq (41.83%) and (41.57%) [137,138]. These apparently contradictory findings can be reconcilable when one considers three interdependent drivers: sampling frame, local strain composition, and detection methods.

Firstly, sampling methods can strongly shape observed prevalence. Collections enriched for invasive, haemolytic or complicated extra-intestinal pathogenic *E. coli* isolates, such as urosepsis, pyelonephritis, or targeted haemolytic strain surveys, will naturally show higher Cytotoxic necrotising factor 1 (CNF1) carriage than unselected outpatient cystitis series or mixed-surveillance cohorts [132]. Secondly, CNF1 is phylogenetically concentrated in particular ExPEC lineages, often B2-associated sequence types; centres with a local dominance of these lineages therefore report inflated CNF1 rates compared with regions where other lineages predominate [139]. Thirdly, methodological choices, PCR primer targets, whether assays screen only *cnf1* versus all *cnf* variants, and the use of whole-genome sequencing versus targeted PCR do change sensitivity and specificity and thus prevalence estimates [132,140]. We recommend that our 9.52% finding be limited to comparison with other studies with comparable sampling frames and detection methods; where possible, they should stratify prevalence by clinical syndrome (CAUTI versus community UTI), phylogroup, and specimen source.

CNF1 is a potent virulence factor that activates Rho GTPases in host cells, leading to cytoskeletal rearrangement, epithelial cell damage, and proinflammatory cytokine release [140,141]. In CAUTIs, which already involve mechanical/physical injury and biofilm formation, even a modest CNF1 prevalence may suggest that a subset of *E. coli* strains can cause deeper tissue invasion and persistent inflammation. Clinical studies have linked CNF1-positive *E. coli* with more severe disease outcomes, such as upper UTIs, bacteraemia, and recurrence [140,142]. Although our cohort shows a relatively low prevalence (9.52%), this indicates that a minority of isolates may contribute disproportionately to morbidity and prolonged hospitalisation, particularly in catheterised adults with compromised mucosal barriers.

There was no observed significant statistical association between *cnf* and the demographic variables.

This study observed a (77.4%) prevalence of the aerobactin (*aer*) gene, which is consistent with studies from Poland (67–70%) [124], Belgium 73% [143], and Iran (70.5%) [34], but discordant studies were also observed in India (57%) and (65%) [144,145], Tunisia (52.2%) [29], Ethiopia (54.50%) [19], and Iran (58.6%) [33]. The higher aerobactin prevalence observed in our study compared with several published cohorts likely reflects differences in the study population and isolate source. Unlike community-based UTI cohorts, where aerobactin frequencies are often lower [29,146], our isolates originated from catheter-associated infections, a setting known to enrich for highly virulent ExPEC lineages with potent iron acquisition systems [143]. Catheter-associated biofilm environments exert strong selective pressure for siderophore-producing strains, which may account for the elevated *aer* carriage [147,148,149]. Additionally, methodological differences in gene targets and detection sensitivity among studies can contribute to apparent discordance [124]. Geographic variation in circulating UPEC lineages may further explain the higher prevalence observed in our setting [145].

Aerobactin is a potent siderophore that improves iron acquisition in the iron-poor urinary tract, promoting bacterial growth, survival under oxidative stress, and persistence. Studies show that aerobactin is enriched in invasive UPEC lineages associated with urosepsis and complicated UTIs [110,150]. Locally, this could potentially translate into a higher proportion of patients at risk of complicated infection, especially those who are older, comorbid or long-term-catheterised [110,150], which is in accordance with to our study, which found 47.69% of all the *aer*-positive samples belonged to patients aged >65 years old. Aerobactin frequently co-occurs with plasmids and ExPEC virulence and resistance determinants. While *aer* itself is not an antibiotic-resistant gene, its common genomic context can make toxin-rich strains more likely to carry resistance determinants, raising the risk of treatment failure or the need for broader empiric therapy [143].

## 5. Conclusions

This study investigated the distribution of uropathogens and the prevalence of virulence genes among *Escherichia coli* isolates from catheter-associated urinary tract infections in Limpopo Province, South Africa, over a one-year period. A total of 215 specimens yielded 255 uropathogen isolates. *Escherichia coli* was the most prevalent uropathogen (32.8% of isolates), followed by *Klebsiella pneumoniae*, *Enterococcus faecalis*, and *Enterococcus faecium*, a distribution consistent with global and regional reports. Female gender and older age (>65 years) were significantly associated with *E. coli* isolation (*p* = 0.025 and *p* < 0.001, respectively). Polymicrobial infections occurred in 28% of *E. coli* cases, with *E. coli* + *E. faecalis* as the predominant co-infection pair.

Molecular characterisation of 84 *E. coli* isolates revealed high prevalence of *fimH* (79.76%) and *aer* (77.38%), with *sfa* (41.67%), *hly* (27.38%), *papC* (26.19%), and *cnf* (9.52%) occurring at lower frequencies. The most common gene combination was *fimH* + *aer*. No statistically significant associations between virulence gene carriage and demographic variables were identified after correction for multiple comparisons.

These findings document the uropathogen profile and UPEC virulence gene frequencies in a Limpopo Province public healthcare context, where such molecular data are limited. The high prevalence of *fimH* and *aer* genes among CAUTI-associated *E. coli* isolates underscores the need for continued molecular surveillance of CAUTI pathogens, reinforcement of catheter-care protocols, and targeted infection prevention strategies in high-risk patient groups. Future studies incorporating antibiotic susceptibility profiling, sequence typing, and gene expression analysis are needed to extend these findings.

## Figures and Tables

**Table 1:** Primers used for UPEC virulence genes.

Virulence Factor	Target Genes	Primer Name	Primer Sequence (5′–3′)	Size of the PCR Product	References
Type 1 fimbriae	*fimH*	*fimH*-f *fimH*-r	5′-AACAGCGATGATTTCCAGTTTGTGTG-3′ 5′-ATTGCGTACCAGCATTAGCAATGTCC-3′	465	[19,29]
P fimbriae	*PapC*	*pap*1 *pap*2	5′-GACGGCTGTACTGCAGGGTGTGGCG-3′ 5′-ATATCCTTTCTGCAGGGATGCAATA-3′	328	[19,29]
S and F1C fimbriae	*Sfa/focDEH region*	*sfa*1 *sfa*2	5′-CTCCGGAGAACTGGGTGCATCTTAC-3′ 5′-CGGAGGAGTAATTACAAACCTGGCA-3′	410	[19,29]
Hemolysin	*HlyCA region*	*hly* s *hly* as	5′-AGATTCTTGGGCATGTATCCT-3′ 5′-TTGCTTTGCAGACTGTAGTGT-3′	556	[19,29]
Cytotoxic necrotizing factor (CNF)	*Cnf*	*cnf* s *cnf* as	5′-TTATATAGTCGTCAAGATGGA-3′ 5′-CACTAAGCTTTACAATATTGA-3′	693	[19,29]
Aerobactin	*aer*	*aer* s *aer* as	5′-AAACCTGGCTTACGCAACTGT-3′ 5′-ACCCGTCTGCAAATCATGGAT-3′	269	[19,29]

**Table 2:** Cycling conditions for each gene adopted from [19,29].

Target Genes	PCR Type	Initial Denaturation	Denaturation (per Cycle)	Annealing	Extension	Final Extension
*fimH*, *papC*, and *sfa*	Multiplex	94 °C—10 min	94 °C—2 min	60 °C—30 s	72 °C—1 min	72 °C—10 min
*Hly*	Single-plex	94 °C—10 min	94 °C—2 min	50 °C—30 s	72 °C—1 min	72 °C—10 min
*Cnf*	Single-plex	94 °C—10 min	94 °C—2 min	45 °C—30 s	72 °C—1 min	72 °C—10 min
*Aer*	Single-plex	94 °C—10 min	94 °C—2 min	55 °C—30 s	72 °C—1 min	72 °C—10 min

**Table 3:** Association of demographic variables with *E. coli* isolation rate and isolation co-infection rate (mixed vs. single infections).

Demographic Variable		*E. coli* Isolation Rate		*p*-Value		Odds Ratio (95% CI)	Total
		Present	Absent	Chi-Square Test	Fisher’s Exact Test		
Gender	Female	56	67	0.025	-	0.523	215
	Male	28	64				
Age	<1	9	30	<0.001	-	-	215
	1–18	6	8				
	19–25	2	8				
	26–45	11	29				
	46–65	15	29				
	>65	41	27				
Race	African	80	128	-	0.436	2.133	215
	White	4	3				

**Table 4:** Association of demographic variables with *E. coli* co-infection rate (mixed vs. single infections).

Demographic Variable		*E. coli* Co-Infection Rate		*p*-Value		Odds Ratio (95% CI)	Total
		Single	Mixed	Chi-Square Test	Fisher’s Exact Test		
Gender	Female	38	18	0.306	-	1.737	84
	Male	22	6				
Age	<1	7	2	0.100	-	-	84
	1–18	6	0				
	19–25	0	2				
	26–45	8	3				
	46–65	9	6				
	>65	30	11				
Race	African	58	22	-	0.574	0.379	84
	White	2	2				

**Table 5:** Predominant gene combinations among *E. coli* isolated in the study.

Rank	Common Gene Combinations	Count (*n*)
1	*fimH* + *aer*	51
2	*fimH* + *sfa*	30
3	*aer* + *sfa*	28
4	*fimH* + *aer* + *sfa*	24
5	*papC* + *aer*	22
6	*fimH* + *aer* + *papC*	17
7	*fimH* + *papC*	17
8	*fimH* + *hly*	15
9	*fimH* + *hly* + *aer*	14
10	*aer* + *sfa* + *hly*	12

## Data Availability

Raw data will be available upon request.

## Abbreviations

The following abbreviations are used in this manuscript:

µL	Microliter
*aer*	Aerobactin gene
ATCC	American Type Culture Collection
Bp	base pairs
CAUTI	Catheter-Associated Urinary Tract Infection
CAUTIs	Catheter-Associated Urinary Tract Infections
CHCA	α-Cyano-4-hydroxycinnamic acid
*cnf*	Cytotoxic Necrotizing Factor gene
DNA	Deoxyribonucleic Acid
dNTPs	Deoxynucleotide triphosphates
ExPEC	Extraintestinal Pathogenic *E. coli*
*fimH*	Type 1 fimbrial adhesion gene
*foc*	F1C fimbriae gene
*hly*	Hemolysin gene
ICU	Intensive Care Unit
IDSA	Infectious Diseases Society of America
*IL*-6	Interleukin-6
*IL*-8	Interleukin-8
MALDI-TOF	Matrix-Assisted Laser Desorption Ionisation–Time of Flight
MDR	Multidrug-Resistant
mPCR	Multiplex Polymerase Chain Reaction
MVP	Magnetic Viral Protocol (KingFisher protocol name)
NC	Negative Control
NHLS	National Health Laboratory Service
NICU	Neonatal Intensive Care Unit
*papC*	Pyelonephritis-associated pili gene
PC	Positive Control
PCR	Polymerase Chain Reaction
RNA	Ribonucleic Acid
*sfa*	S fimbriae gene
SPSS	Statistical Package for the Social Sciences
ST	Sequence Type
TAE	Tris-Acetate-EDTA buffer
TBE	Tris-Borate-EDTA buffer
UPEC	Uropathogenic *Escherichia coli*
USA	United States of America
UTI	Urinary Tract Infection
UTIs	Urinary Tract Infections
WGS	Whole-Genome Sequencing
